# Real-time ultrasound guidance improves neonatal umbilical venous catheter placement efficiency and reduces liver complications

**DOI:** 10.3389/fped.2025.1655820

**Published:** 2025-10-21

**Authors:** Anna Tomaszkiewicz, Piotr Kruczek, Piotr Szymański, Krystian Walczak, Piotr Teplicki, Alina Sobczak, Jan Mazela

**Affiliations:** ^1^ Department of Neonatology, Poznan University of Medical Sciences, Poznan, Poland; ^2^ Department of Neonatology, R. Czerwiakowski Gynecology and Obstetrics Hospital, Kraków, Poland

**Keywords:** umbilical venous catheter, catheter-related complications, neonatal intensive care, central catheters, ultrasound

## Abstract

**Introduction:**

Umbilical venous catheters (UVCs) are essential in neonatal intensive care, yet blind insertion techniques remain common and increase the risk of malposition and liver injury. This study evaluated whether real-time ultrasound guidance improves UVC placement efficiency and safety.

**Methods:**

We retrospectively analysed 305 neonates who underwent ultrasound-guided UVC placement between July 2023 and January 2025 in a tertiary neonatal unit. The primary outcome was successful tip placement in the subdiaphragmatic vestibule; the secondary outcome was acute catheter-related liver injury.

**Results:**

Correct catheter placement was achieved in 86.56% of cases, with a gradual improvement from 83.81% to 89.12% over time. Only one hepatic haematoma was observed, and no other acute complications occurred.

**Discussion:**

Real-time ultrasound guidance significantly enhances the accuracy and safety of UVC insertion in neonates, minimising hepatic injury risk. Further research should assess its role in prolonged catheter monitoring.

## 1 Introduction

UVC catheters are commonly used in neonatal intensive care units to provide parenteral nutrition, deliver intravenous drugs, and obtain blood samples for laboratory tests in the absence of arterial access. Safe use of UVCs requires correct placement of the catheter tip and continuous monitoring for potential complications associated with its presence in the patient's body (1)*.*

Numerous studies have confirmed that ultrasound is a more accurate method for assessing catheter tip position than conventional radiography (2–4).

However, despite the use of ultrasound for the catheter tip localization, in most centres, the procedure itself is still performed blindly. This approach impacts insertion efficiency and increases the risk of complications.

In this study, we aimed to investigate whether real-time intra-procedural ultrasound assessment improves insertion efficiency and reduces acute catheter-related complications associated with navigation and manipulation during placement, particularly liver injury.

## 2 Methods

### 2.1 Patients

This study retrospectively analysed the medical records of patients admitted to a tertiary neonatal unit between July 2023 and January 2025. The study was conducted at a neonatal center in Kraków, Poland, where approximately 1,700 infants are born annually including premature babies and children with congenital defects. The unit also admits neonates referred from other hospitals in the region and serves as a certified training site for specialization in neonatology Inclusion criteria included an attempt to insert a UVC catheter and a minimum hospital stay of three days. The primary indication for UVC placement was the anticipated need for parenteral nutrition for three or more days. Contraindications included omphalocele, gastroschisis, a planned abdominal surgical procedure within the first days of life, and the absence of a ductus venosus, which was either reported prenatally or identified postnatally during neonatal ultrasound prior to the procedure.

### 2.2 UVC insertion procedure

**Figure 1:**
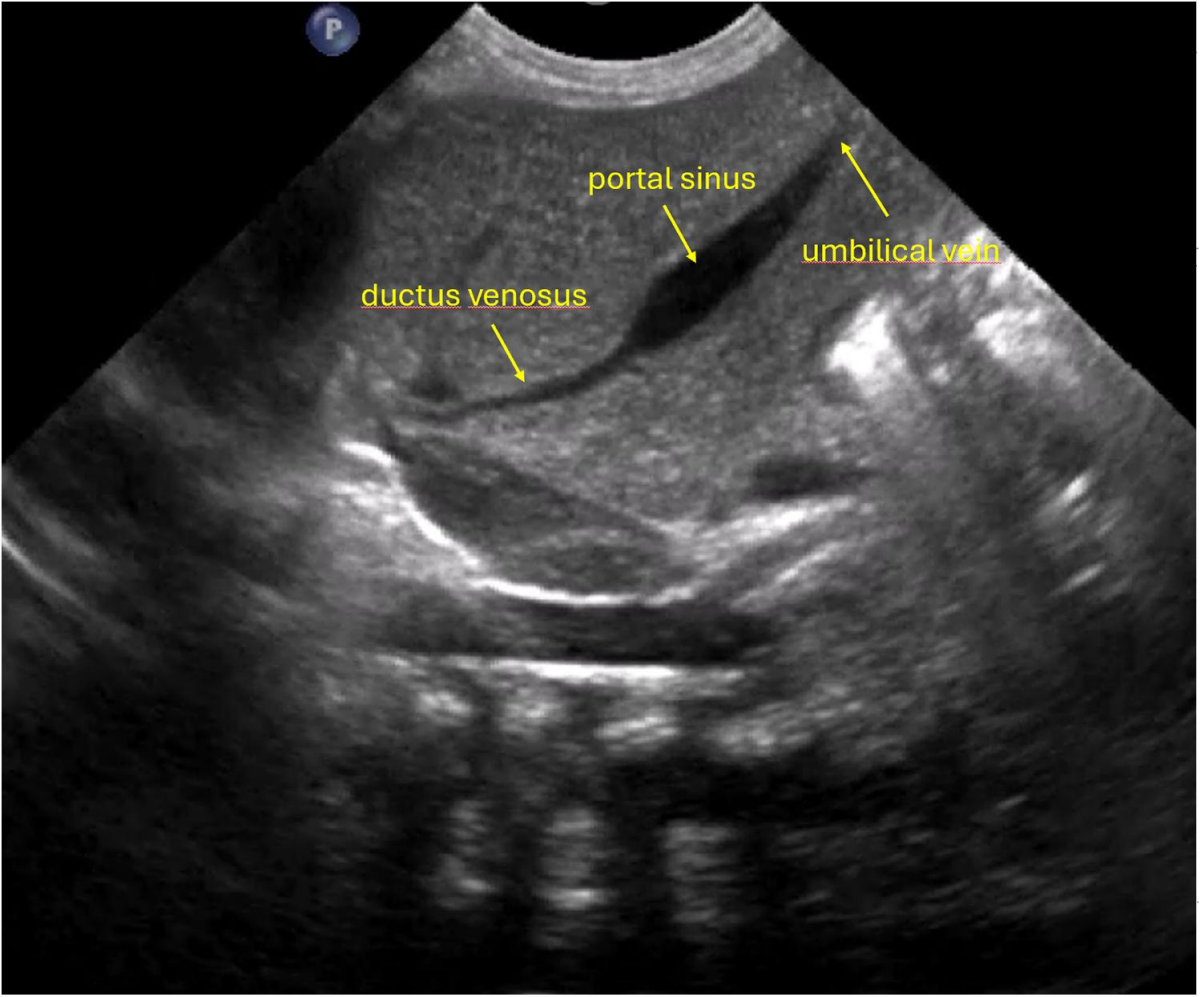
Anatomical configuration characterized by a relatively wider angle between the ductus venosus and the left portal vein, together with a relatively wide ductus venosus lumen, which may facilitate smoother advancement of the catheter during insertion.

**Figure 2:**
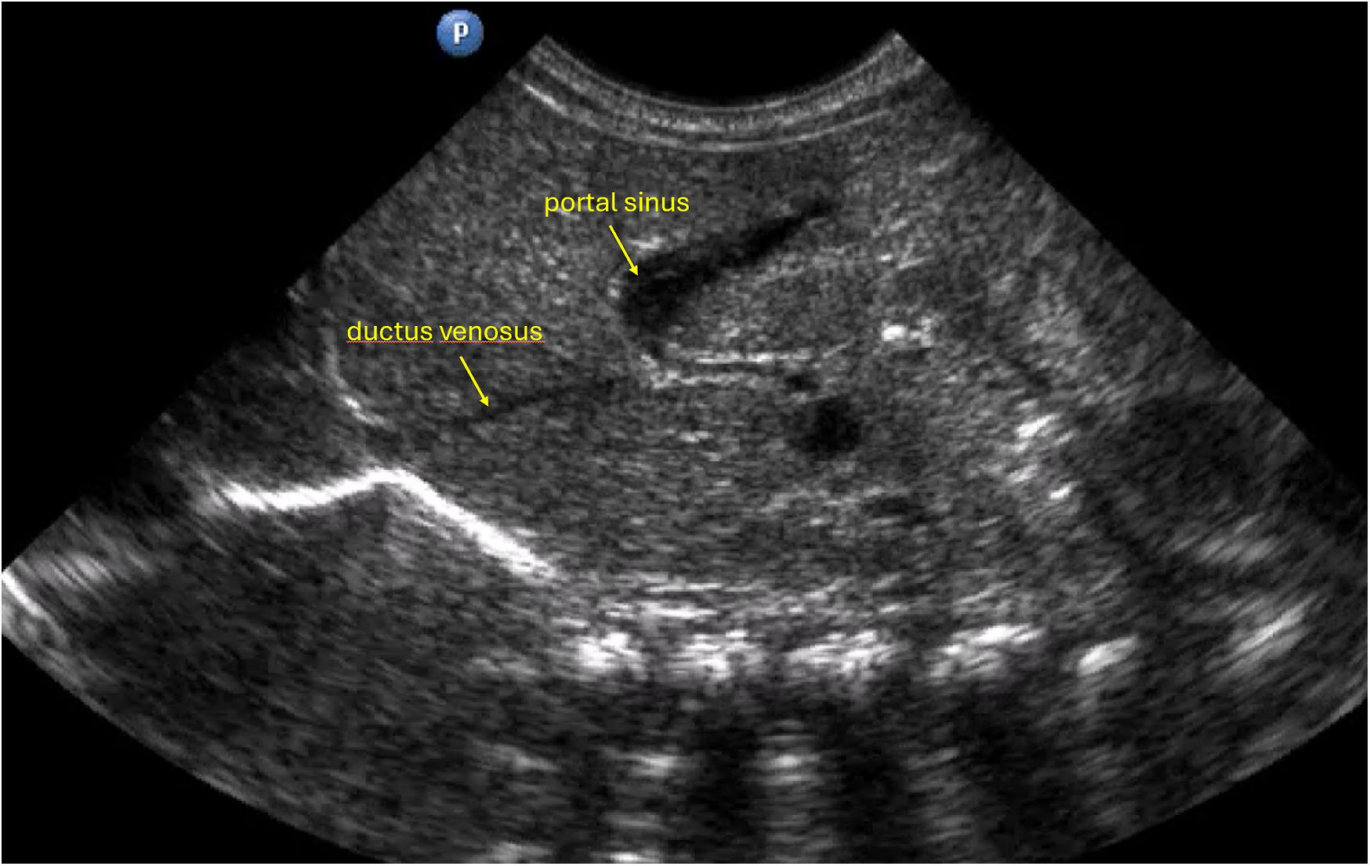
Anatomical configuration in which the ductus venosus arises at a sharp angle and/or presents a narrower lumen, potentially increasing the technical complexity of catheter advancement.

Before UVC insertion, ultrasound was performed to assess the anatomy of the portal tract and the ductus venosus, in order to determine the trajectory of the ductus venosus as it branches from the left portal vein. A wider angle between the ductus venosus and the left portal vein, combined with a wider ductus venosus, facilitated catheter advancement (Figure 1). In this study, a difficult ductus venosus was defined as one with a smaller angle of origin and/or a narrower lumen (Figure 2). Although this was considered a relative contraindication, all patients were included regardless of ductus venosus anatomy.

**Figure 3:**
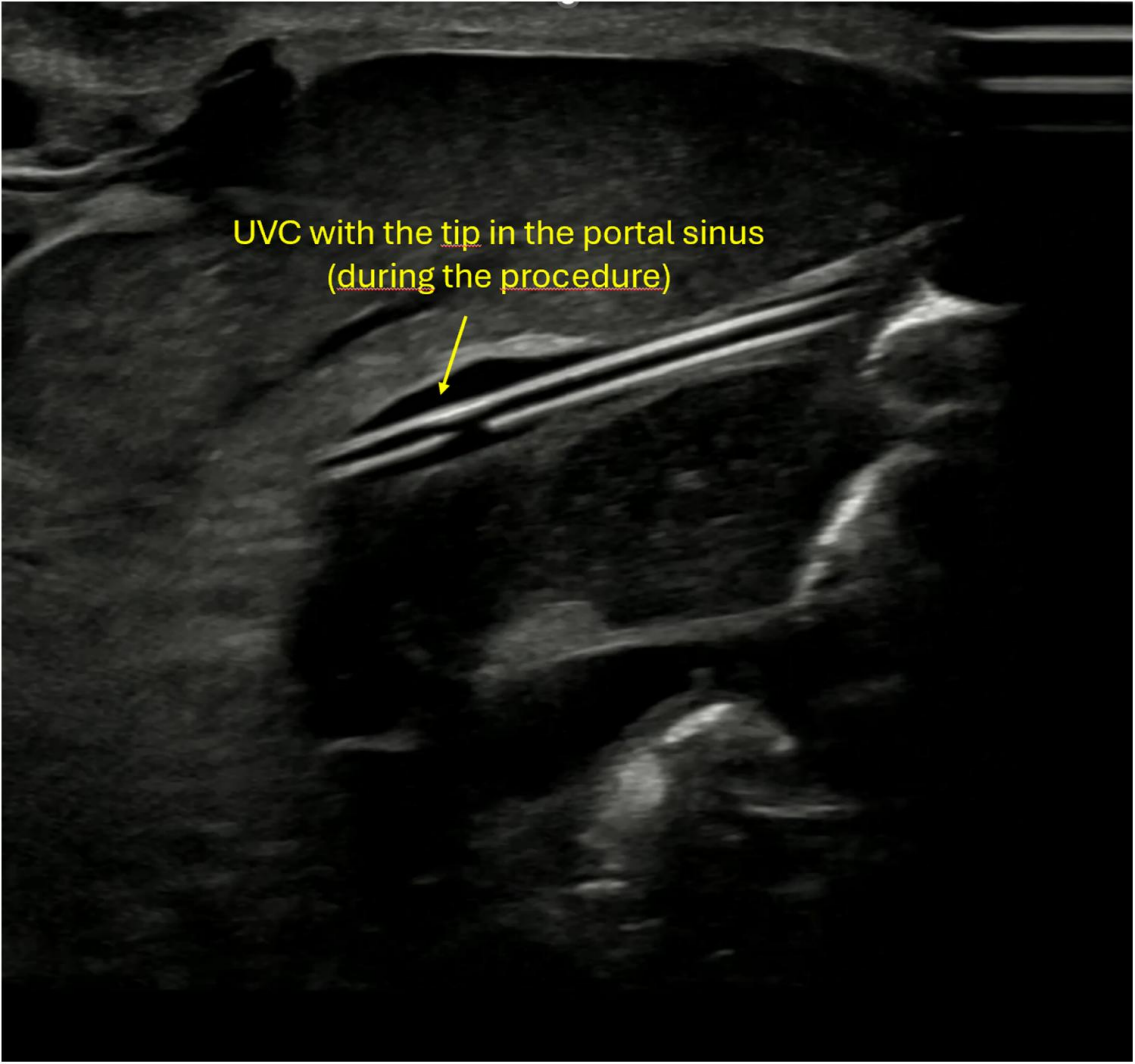
Ultrasound image showing the umbilical venous catheter (UVC) during the procedure, positioned just before entry into the ductus venosus.

**Figure 4:**
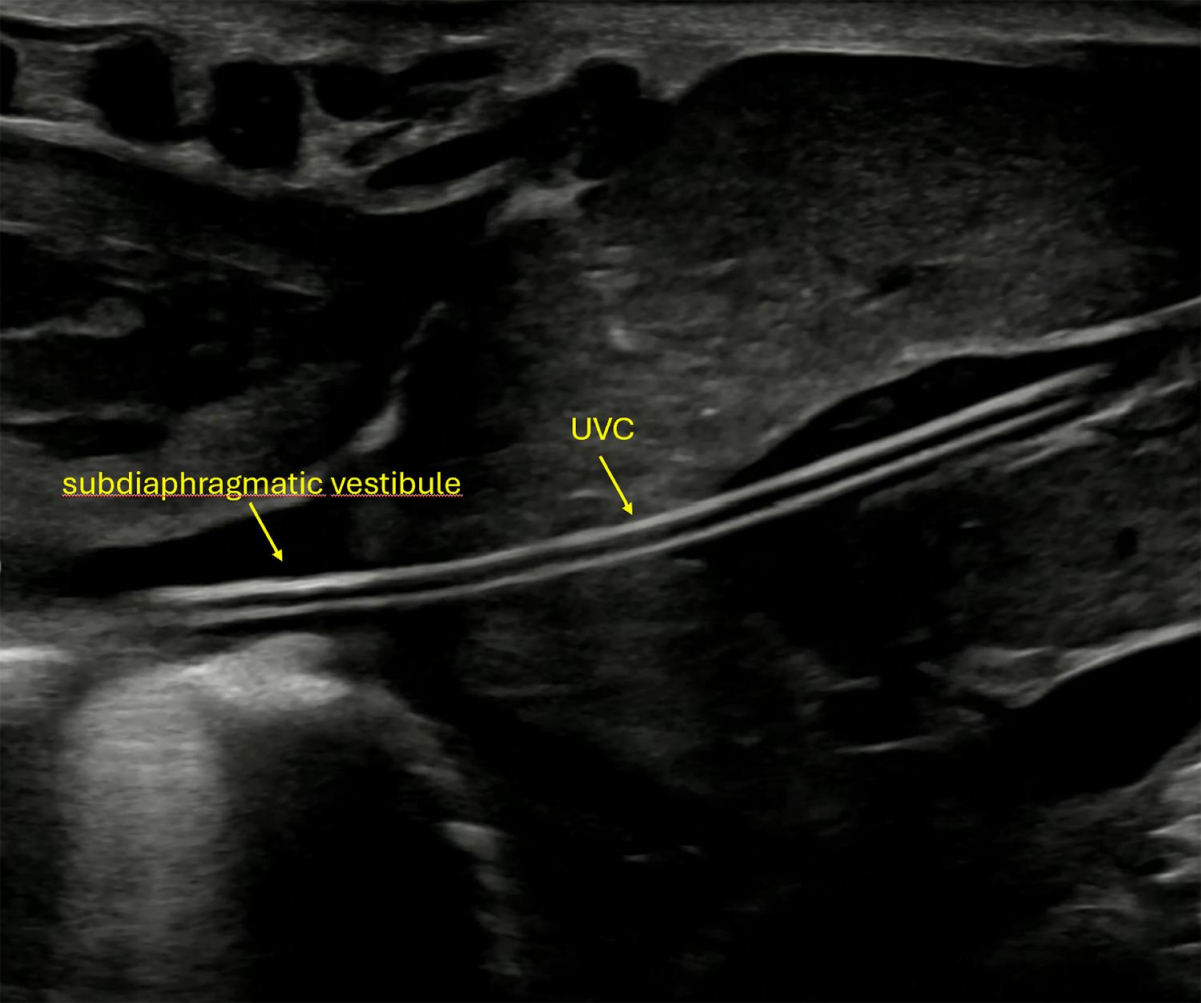
Ultrasound image showing the umbilical venous catheter (UVC) following successful advancement into the ductus venosus, confirming correct positioning.

Two clinicians were involved in each insertion– either a consultant neonatologist and a trained resident, or two trained residents. Residents were considered adequately trained after at least two months of daily ultrasound-guided catheter assessments during procedures or routine monitoring, and after performing a minimum of 25 UVC insertions. After incising the umbilical vein and advancing the catheter approximately 4–5 cm, further positioning was guided by ultrasound. The catheter was first visualized in the umbilical vein and then gradually advanced into the portal sinus under continuous ultrasound guidance, until reaching the ductus venosus. Correct catheter tip placement was defined as positioning in the subdiaphragmatic vestibule. The procedure was completed by securing the catheter. If difficulty arose in directing the catheter through the venous system into the subdiaphragmatic vestibule various manoeuvres were employed to achieve proper placement, such as posterior liver mobilization (5), gentle compression of the upper abdomen—which involved applying titrated pressure to the liver with a transducer in order to compress the left portal vein, accentuate the caudal angulation of the right portal vein, and straighten the “S-turn” from the portal sinus to the ductus venosus, a manoeuvre most frequently used in our practice (6)—or slight rotation of the patient to the right (7). All manoeuvres were performed under continuous ultrasound visualization, with the choice determined by whether the catheter was seen entering the liver parenchyma or deviating into the right portal vein—information available only with real-time ultrasound. For imaging, either a microconvex transducer or a 12–15 MHz linear probe was used. The choice of probe depended primarily on the patient's body weight: the smaller microconvex transducer was used mainly in patients weighing less than 1,500 g. The entire procedure, including the final catheter placement, was recorded. A longitudinal subcostal view was employed to visualize the catheter. Figure 3 shows the UVC during the procedure, prior to entering the ductus venosus, whereas Figure 4 demonstrates the catheter after successful advancement into the ductus venosus.

In the Supplementary Files, we have included a video showing the placement of a UVC under ultrasound guidance.

If initial placement was unsuccessful, the catheter was withdrawn, and an alternative vascular access was required. Ultrasound assessments were conducted every 24 h to monitor for complications until catheter removal. The study's primary outcome was establishing the number of successfully placed catheters. The secondary outcome was the occurrence of mechanical liver injury related to catheter insertion and manipulation excluding thrombosis.

### 2.3 Statistics

The calculations were performed using TIBCO Statistica 13 and PQStat from PQStat Software. The level of significance was set to *α* = 0.05, and the result was considered statistically significant when *p* < *α*.

To examine the relationship between categorical variables, the chi-square independence test or Fisher-Freeman-Halton test was calculated.

The normality of the distribution of variables was tested with the Shapiro–Wilk test. To compare two groups of variables with a normal distribution and equal variance, the Student's *t*-test for unrelated samples was calculated. In the case of non-compliance with the normal distribution or for variables measured on an ordinal scale, the Mann–Whitney test was calculated.

## 3 Results

### 3.1 Study groups

During the study period, ultrasound guided UVC placement was attempted in 310 neonates. Four patients were transferred to another hospital with surgical facilities, and one patient died. Of the transferred cases, three had congenital heart defects requiring cardiac surgery, and one had oesophageal atresia. The infant who died was an extremely premature neonate born at 24 weeks of gestation, who succumbed to congenital sepsis. These cases were excluded from the final analysis due to the inability to assess procedure-related complications. The final analysis included 305 patients.

The statistical analysis showed no differences between the group in which the catheter was successfully inserted and the group in which the catheter was not successfully inserted in terms of gender, body weight, Apgar score at 5 min. The only statistically significant difference was the gestational age. The statistical results and group characteristics are summarized in Table 1.

**Table 1:** The statistical results and group characteristics.

Variable	Successful UVC placement (*n* = 264)	Unsuccessful UVC placement (*n* = 41)	*p* value
Sex (female)	120	18	*p* = 0,853
Birth weight (g), mean ± SD	2746,5 (±703,7)	2956,8 (±747,3)	*p* = 0,0785
Birth weight (g), minimum	860	1,310	
Birth weight (g), maximum	5,010	4,530	
Gestational age (weeks), median	37	38	*p* = 0,019
Gestational age (weeks), maximum	41	41	
Gestational age (weeks), minimum	27	30	
Apgar score (5 min), median	10	10	*p* = 0,745

The analysis of the reasons for catheter insertion took into account the most common ones, i.e., respiratory failure, prematurity, low body weight, hypoglycaemia and congenital anomalies (Table 2) and showed no statistical difference between the groups: *p* = 0.130.

**Table 2:** The reasons for catheter insertion.

Reason for UVC placement	Number of patients	Percentage
Respiratory failure	82	26,89
Low birth weight	34	11,15
Prematurity	97	31,80
Hypoglycaemia	26	8,52
Congenital defects	17	5,57
Others	49	16,07

### 3.2 Succes rate and complications

The overall success rate for correct placement of UVC was 86.56% and additional analysis over a six-month period revealed an increasing trend in insertion efficiency (Table 3).

**Table 3:** Efficacy of catheter insertion.

Period	Number of patients	Number of UVC	Efficacy
**01.07.23–31.12.24**	**305**	**264**	**86,56%**
01.07.23–31.12.23	105	88	83,81%
01.01.24–30.06.24	99	86	86,87%
01.07.24–31.12.24	101	90	89,12%

Bold text indicates effectiveness over the whole study period; effectiveness for 6-month periods are shown below.

However, using the Cochran–Armitage trend test, this difference did not reach statistical significance (*p* = 0.2645). Analysis of catheter insertion complications identified one hepatic hematoma around the left portal vein branch, detected via ultrasound 24 h post-insertion. In this case, the catheter tip was correctly positioned. No other liver complications were observed.

## 4 Discussion

The 2020 guidelines of the European Society of Anaesthesiology (ESA) emphasize “the global use of ultrasound”, which formed the basis of this study, namely “recognition of possible local disease; verification of the direction of guidewires and catheters in the vessel, verification of the correct position of the catheter tip; detection of possible postprocedural early and late complications” (8). These principles are incorporated into our daily practice to improve catheter insertion efficiency and minimize procedure-related complications.

Pittiruti et al. stated that “radiological methods (fluoroscopy, chest x-ray) no longer play any role in the insertion of vascular accesses in neonates and children” (9), as supported by recent studies. In our study, we did not compare ultrasound with x ray because ultrasound is the sole method used in our department for assessing central catheters. Given the existing literature, routine x-ray assessment no longer appears justified. In all cases, we successfully visualized the catheter tip using ultrasound. We have achieved a high UVC placement efficiency in comparable to the large study by D'Andrea (10) and to the smaller survey conducted by Kishigami et al. (6), demonstrating that real-time tip navigation significantly increases the likelihood of correct primary catheter positioning. Similar results and insertion techniques were reported by Kozyak (11). However, a randomized controlled trial conducted by Mishra (12) failed to show a statistically significant difference between ultrasound-guided and blind UVC insertion. It is worth noting that their study reported a much lower catheter placement efficiency compared to our findings and those of D'Andrea (10).

Additionally, our study observed an increasing trend in the catheter insertion efficiency. Our centre is a resident training facility, and in each case, a resident assisted with the catheter placement. This aligns with findings from Rubortone (13), who demonstrated that ultrasound guidance and structured training improve the success rate of UVC placements. Although we noted a temporal increase in efficiency, the differences between the analysed time periods were not statistically significant.

Ultrasound-guided catheter positioning not only reduces malpositioning but also shortens procedure time, as confirmed in an randomized controlled trial by Kaur et al. (14). This efficiency may represent another significant advantage of this approach.

While we did not record procedure duration in our study, this represents a likely additional benefit of the technique.

The differences between successful and unsuccessful UVC groups suggest that catheter placement is more challenging in more mature neonates. We observed that the ductus venosus (DV) tends to be narrower in these infants.

In our study, DV anatomy was assessed prior to each procedure, and our observations indicate that beyond gestational age—which was statistically significant—the configuration of the DV has a substantial impact on the success of ultrasound-guided catheterization. However, further research is needed to objectively determine which anatomical variants of the DV make catheter placement particularly difficult. Such a detailed anatomical analysis was beyond the scope of this study.

The secondary endpoint of our study was early complications related to catheter insertion, particularly liver injury. Hematomas and thrombosis may occur if the catheter is inadvertently advanced into the liver parenchyma or becomes malpositioned (15). Although all catheters in our study were correctly positioned, various facilitating manoeuvres were employed to achieve optimal placement. We aimed to evaluate the safety of these manoeuvres, as inadvertent advancement into the liver parenchyma is sometimes unavoidable when the catheter initially follows an incorrect course rather than the ductus venosus. Without real-time visualization, the operator may remain unaware of such complications. Our findings suggest that, provided insertion is performed under continuous ultrasound guidance, the risk of liver injury is minimal. Data on complication rates prior to the introduction of ultrasound guidance are not available, as this method has been routinely employed in our department for many years. This study therefore summarizes outcomes from the most recent period of our practice. We did not assess late complications such as thrombosis, which we considered unrelated to the insertion technique. Importantly, no catheter-related infections attributable to the insertion technique (i.e., within 72 h of catheter placement) were observed in our cohort.

Major strengths of our study are: large number of patients included in the analysis, prospectively established guideline for ultrasound guided UVC positioning.

The primary limitation of our study is that it was conducted in a single centre and utilized a retrospective design.

In conclusions, our study showed that ultrasound guided UVC placement is feasible, effective, safe and decrease number of potential complications. Further study is needed to establish if continuous ultrasound monitoring of UVC can allow for safe and longer utilization of such vascular catheters.

## Data Availability

The raw data supporting the conclusions of this article will be made available by the authors, without undue reservation.
